# Utility of modified Rankin Scale for brain vascular malformations in hereditary hemorrhagic telangiectasia

**DOI:** 10.1186/s13023-021-02012-y

**Published:** 2021-09-19

**Authors:** K. P. Thompson, J. Nelson, H. Kim, S. M. Weinsheimer, D. A. Marchuk, M. T. Lawton, T. Krings, M. E. Faughnan, Brain Vascular Malformation Consortium HHT Investigator Group, Murali Chakinala, Marianne S. Clancy, Marie E. Faughnan, James R. Gossage, Steven W. Hetts, Vivek Iyer, Raj S. Kasthuri, Helen Kim, Timo Krings, Michael T. Lawton, Doris Lin, Hans-Jurgen Mager, Douglas A. Marchuk, Justin P. McWilliams, Jamie McDonald, Ludmilla Pawlikowska, Jeffrey Pollak, Felix Ratjen, Karen Swanson, Dilini Vethanayagam, Shantel Weinsheimer, Andrew J. White, Pearce Wilcox

**Affiliations:** 1 Toronto HHT Centre, St. Michael’s Hospital and Li Ka Shing Knowledge Institute, Toronto, Canada; 2 Division of Respirology, Department of Medicine, University of Toronto, Toronto, Canada; 3 Center for Cerebrovascular Research, Department of Anesthesia and Perioperative Care, University of California, San Francisco, CA USA; 4 Institute for Human Genetics, University of California, San Francisco, CA USA; 5 Department of Epidemiology and Biostatistics, University of California, San Francisco, CA USA; 6 Department of Molecular Genetics and Microbiology, Duke University Medical Center, Durham, NC USA; 7 Department of Neurosurgery, Barrow Neurological Institute, Phoenix, AZ USA; 8 Division of Neurosurgery, Department of Medical Imaging, Department of Surgery, University of Toronto, Toronto, Canada; 9 Division of Neuroradiology, Toronto Western Hospital, Univeristy Health Network, Toronto, Canada

## Abstract

**Background:**

Approximately 10% of hereditary hemorrhagic telangiectasia (HHT) patients harbour brain vascular malformations (VMs). Intracranial hemorrhage (ICH) from brain VMs can lead to death or morbidity, while treatment options for brain VMs also have associated morbidity. The modified Rankin Scale (mRS) may provide an approach to identifying HHT-brain VM patients with poor outcomes, and their predictors. We aimed to measure the relationship between mRS score and brain VM, brain VM number, as well as other aspects of HHT, at enrollment and during prospective follow-up.

**Methods:**

1637 HHT patients (342 with brain VMs) were recruited from 14 HHT centres of the Brain Vascular Malformation Consortium since 2010 and followed prospectively (mean = 3.4 years). We tested whether the presence of brain VM, other HHT organ involvement, and HHT mutation genotype were associated with worse mRS scores at baseline and during follow-up, using linear mixed models, adjusting for age, sex, and year of visit.

**Results:**

Presence of brain VMs was not associated with worse mRS score at baseline and there was no significant worsening of mRS with prospective follow-up in these patients; 92% had baseline mRS of 0–2. HHT-related gastrointestinal (GI) bleeding was associated with worse mRS scores at baseline (0.37, 95% CI 0.26–0.47, *p* < 0.001), as were history of anemia (0.35, 95% CI 0.27–0.43, *p* < 0.001) and liver VMs (0.19, 95% CI 0.09–0.30, *p* < 0.001). Presence of pulmonary arteriovenous malformations (AVMs) was not associated with worse mRS scores at baseline. mRS score was not associated with either HHT genotype (*Endoglin* vs *ACVRL1*). Only GI bleeding was associated with a significantly worsening mRS during prospective follow-up (0.64, 95% CI 0.21–1.08, *p* = 0.004).

**Conclusion:**

Most HHT-brain VM patients had good functional capacity (mRS scores 0–2) at baseline that did not change significantly over 3.4 mean years of follow-up, suggesting that mRS may not be useful for predicting or measuring outcomes in these patients. However, HHT patients with GI bleeding, anemia history or liver VMs had worse mRS scores, suggesting significant impact of these manifestations on functional capacity. Our study demonstrates the insensitivity of the mRS as an outcomes measure in HHT brain VM patients and reinforces the continued need to develop outcomes measures, and their predictors, in this group.

## Introduction

Brain vascular malformations (VMs) remain a therapeutic and prevention challenge in hereditary hemorrhagic telangiectasia (HHT), present in approximately 10% of HHT patients, across all ages and all HHT genotypes [1–3]. Intracranial hemorrhage (ICH) from brain VMs can be fatal and can occur in children and adults with HHT [4], leading to death or morbidity and disability. Available treatment modalities for brain VMs are invasive and their associated morbidity must be weighed against ICH risk and brain VM-related morbidity. Despite growing evidence for antiangiogenic and pathway therapies to treat other organ involvement in HHT [5], there are no approved medical therapies for brain VMs in these patients. Though the overall annual rate of ICH from brain VMs in HHT is relatively low, the distribution is wide, with a large range of risk [6]. One high-risk sub-group consisting of HHT-brain VM patients with previous ICH has been previously identified [6]. Finally, brain VM phenotypes in HHT, including common lesion multiplicity, as well as lesion types defined on imaging, have also been previously characterized, though their association with ICH risk remains unquantified [2, 7–9]. Confirming additional predictors of ICH remains limited by the overall low ICH rate and the rarity of HHT-brain VMs, even for our multicenter network with the largest HHT-brain VM cohort in the world, and clinical decision-making remains problematic.

Functional disability scores, such as the modified Rankin Scale (mRS), may provide an additional approach to identifying HHT-brain VM patients with poor outcomes, and their predictors. The modified Rankin Scale is commonly used to measure disability of people who have suffered a stroke or other cause of neurological disability [10–12], and is also widely applied as an end point for clinical trials [10, 12]. The 7-point scale encompasses the entire range of functional outcomes from no symptoms to death, its categories are intuitive for both clinicians and patients, and its validity has been demonstrated by its strong association with measures of stroke pathology and other stroke scales [13].

Though few studies to date have reported on mRS in HHT-brain VM patients exclusively [14, 15], there are several that have reported on mRS in brain VM patients in general and have used the mRS to validate brain VM grading scales. In one study, the mRS was used to assess outcomes after brain AVM radiosurgery and it was found that the radiosurgery-based AVM score correlated with a worsening mRS after treatment [16], suggesting a relationship between baseline grading of brain AVM severity and an increasing (worsening) mRS score across time. The Supplemented Spetzler-Martin grade (Supp-SM), a modified brain AVM grading scale which aims to predict neurologic outcomes after surgery, was developed based on the association between predictor variables and change in preoperative and postoperative mRS scores in 300 patients that underwent AVM microsurgery [17]. Further, in a study on treatment outcomes of unruptured AVMs, mRS score deterioration, permanent deficits, and impaired functional outcome occurred less frequently in Spetzler-Martin grade I and II patients compared with grade III to V patients [18], pointing to a relationship among baseline grade I and II brain AVM patients, low baseline mRS score, and better long-term functional outcomes. Several other studies have employed the mRS to assess neurological outcomes after surgery for brain AVM, with similar findings [19–21].

Reports on mRS in HHT patients with brain VMs remain limited. Our group previously reported retrospective data comparing mRS scores after surgical vs nonsurgical management of brain AVMs in HHT patients, demonstrating that patients treated surgically for brain AVMs had similar long-term functional scores to those with nonsurgical therapy, with fewer unfavourable outcomes [14]. Another group aiming to clarify the clinical characteristics and hemorrhagic risk of HHT-related brain AVMs found that patients with HHT presented with better mRS, as compared to patients with sporadic AVMs [15].

Given the risk of ICH and the morbidity associated with treatment options for HHT-brain VM patients, there is a critical need to expand brain VM outcome measures and predictors, including mRS and its change over time. We aimed to measure the relationship between mRS score and presence of brain VM, brain VM number, and other aspects of HHT, including HHT genotype and organ phenotype, as well as how mRS score changes over time in relation to these factors.

## Methods

1687 HHT patients were enrolled by the Brain Vascular Malformation Consortium (BVMC, https://www.rarediseasesnetwork.org/cms/bvmc/) at multiple recruiting centers in the US, Canada and the Netherlands between 2010 and 2020. Cohort recruitment has been previously described [22]. All patients provided written informed consent. The study protocol was approved by the institutional review board at each recruiting centre. Patients were screened for organ VMs and other clinical features as part of their routine clinical care according to standard clinical practice [23] and International HHT Guidelines [24, 25], including: comprehensive history, physical, routine blood tests, screening for pulmonary AVM by contrast echocardiography (in adults and children), brain VM screening by magnetic resonance imaging (in adults and children), clinical screening for liver VM (assessment for chronic right upper quadrant pain, portal hypertension, high-output heart failure, liver bruit on examination, abnormal liver function tests) and clinical screening for recurrent spontaneous epistaxis (> 1 episode per month for > 1 year), and HHT-related GI-bleeding (anemia, iron deficiency, known GI telangiectases on endoscopy, melena, rectal bleeding). As part of their routine clinical care, if screening was positive for pulmonary AVM or brain VM, patients underwent further diagnostic imaging and treatment, where appropriate. If clinical assessment was suggestive of symptomatic liver VM, diagnostic imaging was recommended and therapy where appropriate. If initial clinical assessment was suggestive of HHT-related GI bleeding, then diagnostic endoscopy was recommended, and endoscopic, medical and supportive therapies were undertaken on a case-by-case basis. The BVMC HHT cohort targets 25% brain VM-positive patients; other characteristics are similar to other cohorts [25, 26]. Thus, we have a higher proportion of HHT brain VM patients than other published cohorts by design. This sampling design, however, should not create or exacerbate an existing bias when measuring differences between brain VM and non-brain VM HHT patients.

The analysis sample included 1637 HHT patients (342 with brain VMs) with a mRS value recorded at enrollment. We calculated the median and interquartile range of age at enrollment for the entire sample and stratified by mRS (0–2 vs. 3–5). Similarly, we computed counts and percentages for categorical variables. We used linear mixed models to assess whether mRS (the outcome) was associated with various HHT clinical manifestations. We tested each clinical manifestation individually. These models estimated coefficients for both the effect of the clinical manifestation on mRS at enrollment and the interaction of the clinical manifestation with the year of the visit (i.e. the change of mRS through time). Models adjusted for age at enrollment, the year of the visit, and sex. These models included random intercepts for patients and random slopes for patients through time. Data analysis was conducted using Stata 15.1 SE software (StataCorp. 2017. *Stata Statistical Software: Release 15*. College Station, TX: StataCorp LLC). We considered p-values less than 0.05 to be statistically significant.

## Results

A total of 1687 patients were enrolled in the study between May 2010 and January 2020, of which 1637 (97%) had a baseline mRS value recorded and were included in the analysis sample. In the analysis sample, 1258 (77%) had at least one follow-up mRS recorded. A total of 3601 annual follow-up visits with mRS values were logged (an average of 2.20 visits per patient). The mean follow-up time was 2.64 years; the longest follow-up time was 10 years.

Summary statistics are provided in Tables 1 and 2. Most patients were functionally independent at enrollment (95% with an mRS of 0 to 2). Proportions of patients, by organ manifestation/genotype, with mRS >  = 3, are detailed in Table 3, with 8% of brain VM patients having an mRS >  = 3. The median age was 49 (IQR: 33–60). Females comprised 59% of the sample. Of those with mutation information available, 55% had *Endoglin* mutations, 42% had *ACVRL1* mutations, and 3% had a *SMAD4* mutation. With respect to the prevalence of clinical manifestations, 21% had brain VM, 50% had pulmonary AVM, 17% had liver VMs, 17% had GI bleeding, and 48% were anemic. Within the HHT brain VM population reported here, 135/335 (40.3%) patients had a symptomatic presentation of the brain VM. Amongst those with a symptomatic presentation, 46/133 (34.6%) had presented with ICH. There was no difference in mRS score at enrollment between patients who had been symptomatic at brain VM diagnosis, compared to those who were asymptomatic of brain VM at diagnosis (screened patients): 120/135 (88.8%) initially symptomatic brain VM patients had an mRS of 0–2, similar to 187/200 (93.5%) of initially asymptomatic patients. Likewise, 15/135 (11.1%) initially symptomatic patients had an mRS of >  = 3, compared to 13/200 (6.5%) initially asymptomatic patients.

**Table 1:** Summary statistics in 1637 patients

Characteristic	mRS 0–2 (n = 1544)	mRS 3 + (n = 93)	Overall (n = 1637)
Age at enrollment	48 (32.5–59)	56 (43–64)	49 (33–60)
Female	921 (60%)	52 (56%)	973 (59%)
Mutation			
* ACVRL1*	457/1081 (42%)	18/52 (35%)	475/1133 (42%)
* Endoglin*	588/1081 (54%)	31/52 (60%)	619/1133 (55%)
* SMAD4*	35/1081 (3%)	3/52 (6%)	38/1133 (3%)
Brain VM	314/1537 (20%)	28/92 (30%)	342/1629 (21%)
Pulmonary AVM	737/1488 (50%)	51/90 (57%)	788/1578 (50%)
Liver VM	239/1469 (16%)	28/91 (31%)	267/1560 (17%)
GI bleeding	230/1483 (16%)	34/91 (39%)	264/1571 (17%)
Anemia	688/1490 (46%)	63/88 (72%)	751/1578 (48%)

Values are median (interquartile range), n (%), or n/total (%)

**Table 2:** Modified Rankin Scale at enrollment, n = 1637

Value	n (%)
0—No symptoms	222 (14%)
1—No significant disability despite symptoms	767 (47%)
2—Slight disability	555 (34%)
3—Moderate disability	73 (4%)
4—Moderately severe disability	17 (1%)
5—Severe disability	3 (< 1%)
*6—Death is not included as deceased patients were excluded

**Table 3:** Summary statistics in patients with Modified Rankin Score of 3 or greater

Characteristic	Proportion with mRS 3 +	Median (range)
Mutation		
* ACVRL1*	18/475 (4%)	1 (0–5)
* Endoglin*	31/619 (5%)	1 (0–4)
*SMAD4*	3/39 (8%)	1 (0–4)
Brain VM	28/342 (8%)	1 (0–5)
Pulmonary AVM	51/788 (6%)	1 (0–5)
Liver VM	28/267 (10%)	1 (0–5)
GI bleeding	34/264 (13%)	1 (0 –5)
Anemia	63/751 (8%)	1 (0–5)

Value in mRS 3 + column are n/total (%)

Results for the linear mixed model analyses are shown in Table 4. We found that the presence of brain VM, and brain VM number, were not associated with worse mRS scores at baseline. Amongst brain VM patients with available data, the average number of VMs was 1.76 (median = 1, max = 10). 53.5% of the VMs were AVMs, 38.5% were capillary malformations / micro-AVMs, and 8.0% were arteriovenous fistulas (AVFs). There was no association between HHT genotype and worse mRS scores. Several other clinical manifestations were associated with worse mRS scores at baseline. Patients with GI bleeding had mRS values 0.37 units higher at baseline compared to those without (95% CI 0.26–0.47, *p* < 0.001). Similarly, mRS values were worse at baseline for patients with history of anemia (0.35, 95% CI 0.27–0.43, *p* < 0.001) and liver VMs (0.19, 95% CI 0.09–0.30, *p* < 0.001). Only GI bleeding was associated with significant change in mRS score over prospective follow-up; GI bleeders were estimated to have worsened by 0.64 points per 10 years after enrollment (95% CI 0.21–1.08, *p* = 0.004). We found that patients that died (mRS = 6) during follow-up drove this association, as excluding those patients from the analysis reduced the coefficient estimate to − 0.11 (95% CI − 0.43–0.20, *p* = 0.490).

**Table 4:** Linear mixed regression results

Characteristic	mRS difference at baseline			10 year mRS change		
	Coefficient	95% CI	*p* value	Coefficient	95% CI	*p* value
Age at enrollment (per decade)	0.11	(0.09, 0.13)	< 0.001	n/a	n/a	n/a
Year of visit	n/a	n/a	n/a	0.48	(0.31, 0.64)	< 0.001
Female	0.04	(− 0.04, 0.11)	0.348	− 0.02	(− 0.05, 0.02)	0.310
*ACVRL1* (compared to *Endoglin*)	− 0.01	(− 0.98, 0.77)	0.812	− 0.28	(− 0.11, 0.66)	0.158
Brain VM	− 0.03	(− 0.13, 0.06)	0.506	0.00	(− 0.39, 0.38)	0.981
Pulmonary AVM	0.04	(− 0.04, 0.11)	0.361	0.00	(− 0.35, 0.34)	0.984
Liver VM	0.19	(0.09, 0.30)	< 0.001	0.16	(− 0.29, 0.60)	0.483
GI bleeding	0.37	(0.26, 0.47)	< 0.001	0.64	(0.21, 1.08)	0.004
Anemia	0.35	(0.27, 0.43)	< 0.001	0.34	(0.00, 0.68)	0.051

## Discussion

Presence of brain VMs and brain VM number do not appear to be associated with worse mRS scores at baseline or over time in our study and therefore we have not demonstrated its utility as an outcomes predictor or outcomes measure for HHT patients with brain VMs. We observed that the majority of brain VM patients were functionally independent at enrollment, meaning they had a mRS score between 0 and 2. Only 8% of brain VM patients had an mRS >  = 3, which likely limits our power to detect a mRS association for brain VMs in HHT patients, as few patients on the more severe end of the disability spectrum were included in the analyses.

Although the mRS is used widely as an outcome measure in stroke research [10–12], there have been limitations raised in stroke research as well. For example, a systematic review of interobserver studies demonstrates potentially significant interobserver variability associated with the modified Rankin Scale [27]. Others have highlighted the subjective determination between categories and the reproducibility of the score by examiners and patients as additional limitations of the mRS [28]. It has further been noted that the mRS may be less responsive to change than some other scales given its limited number of levels [29], all of which may impact the usefulness of the mRS in other diseases, including HHT.

Despite the negative results in brain VMs, mRS scores do provide a simple assessment of general functional status that can be useful in HHT. We demonstrate that GI bleeding and history of anemia are associated with worse mRS scores at baseline, in the linear mixed regression model. We also demonstrate that GI bleeding has a significant worsening effect on mRS through time. It has been previously demonstrated that HHT patients with GI bleeding experience significant morbidity, requiring more transfusions and more emergency room and hospital admissions than other HHT patients [30]. Additionally, we recently reported that chronic GI bleeding is associated with increased mortality in HHT patients [31], in keeping with our observed higher mRS scores associated with this HHT manifestation. Further, a report on acute ischemic stroke patients (not HHT) demonstrated that patients who developed GI bleeding had a higher rate of mortality and disability (mRS grade greater than or equal to 4) than those who did not [32]. Others have shown that GI bleeding in stroke patients is associated with neurologic deterioration, in-hospital mortality, and poor functional outcome [33], and patients with anemia have an increased risk of mortality from acute stroke [34]. Several others have reported similar relationships between these manifestations and reduced functional capacity in stroke patients [35, 36], in keeping with our observation that GI bleeding is associated with worse functional outcomes in the HHT population.

We also demonstrate that presence of liver VMs is associated with worse mRS scores at baseline, in the linear mixed regression model. As described in the methods, liver VM patients reported in this series are symptomatic cases. Serious complications of liver VMs have been previously reported and include high-output heart failure, portal hypertension, and biliary disease [37]. Moreover, in an HHT longitudinal study, 25% of liver VM patients experienced liver VM related complications [38], supporting our observed worse longitudinal outcomes and higher mRS scores for patients with liver VMs. Further, it has been demonstrated that liver disease is associated with worse hospital discharge disposition and in-hospital mortality after stroke [39], and patients with liver disease experience more severe strokes and worse outcomes [40], in keeping with our observation that liver VMs are associated with worse functional outcomes in the HHT population.

Presence of pulmonary AVMs is not associated with worse mRS scores in the HHT patient population reported here. Serious complications, such as stroke, brain abscess and pulmonary hemorrhage have been associated with untreated pulmonary AVMs [41], though current practice is for routine asymptomatic screening and preventative management of pulmonary AVMs [23], since at least 2009 with publication of the first HHT Guidelines [24], and therefore the absence of association with poor functional scores is expected.

We also did not observe an association between HHT genotype (*Endoglin* vs *ACVRL1* mutation) and mRS score at baseline or through time, even though pulmonary AVMs and brain VMs have been shown to be more common in *Endoglin* mutation carriers, and liver VMs to be more common in *ACVRL1* mutation carriers [42]. This suggests that while organ-specific involvement is associated with genotype, overall degree of disability, at least measured by mRS, may not differ according to HHT genotype.

Our study has a few limitations. First, the majority of brain VM patients included in the analyses had a mRS score between 0 and 2. This may have limited our power to detect an mRS association for brain VMs, as few patients on the severe end of the disability spectrum were included in the analyses. In addition, genetic information was not available for all patients included in the study, which may have limited our ability to detect an association between HHT genotype and mRS score. Similarly, detailed treatment information was not available for all patients included in the study, and was therefore excluded from the analyses. Correlating mRS with treatment of all aspects of HHT in the future may be beneficial. Lastly, lesional details regarding the severity of brain VMs, liver VMs, and pulmonary AVMs were not available for this study, other than brain VM number, meaning that the analyses were performed based on the presence or absence of these features. Though this may have led us to underestimate the association between brain VMs and mRS score, despite this, we did detect significant associations for liver VMs, GI bleeding, and anemia, supporting the validity of these study results. While this indicates significant impact of these manifestations on functional capacity, we are not recommending the mRS for assessing these aspects of HHT. Further studies, with lesional details such as size and localization, will be helpful.

## Conclusion

Most HHT-brain VM patients reported low mRS scores (0–2) and the mRS did not change significantly over 3.4 mean years of follow-up, suggesting that the mRS may not be useful for predicting outcomes in these patients. However, HHT patients with GI bleeding, history of anemia or liver VMs had worse mRS scores, indicating significant impact of these manifestations on patients’ functional capacity. Our findings suggest the mRS is not a sensitive predictor of outcomes in HHT-brain VM patients and reinforce the ongoing need to identify other predictors of outcomes in this high-risk group.

## Declarations

### Availability of data and materials

All data generated or analyzed during this study are included in this published article.
